# Management of Displaced Midshaft Clavicle Fractures with Figure-of-Eight Bandage: The Impact of Residual Shortening on Shoulder Function

**DOI:** 10.3390/jpm12050759

**Published:** 2022-05-07

**Authors:** Carlo Biz, Davide Scucchiari, Assunta Pozzuoli, Elisa Belluzzi, Nicola Luigi Bragazzi, Antonio Berizzi, Pietro Ruggieri

**Affiliations:** 1 Orthopedics and Orthopedic Oncology, Department of Surgery, Oncology and Gastroenterology DiSCOG, University of Padova, Via Giustiniani 3, 35128 Padova, Italy; carlo.biz@unipd.it (C.B.); davide.scucchiari@gmail.com (D.S.); elisa.belluzzi@unipd.it (E.B.); pietro.ruggieri@unipd.it (P.R.); 2 Musculoskeletal Pathology and Oncology Laboratory, Department of Surgery, Oncology and Gastroenterology, University of Padova, Via Giustiniani 3, 35128 Padova, Italy; 3 Laboratory for Industrial and Applied Mathematics (LIAM), Department of Mathematics and Statistics, York University, Toronto, ON M3J 1P3, Canada; robertobragazzi@gmail.com

**Keywords:** clavicle fracture, midshaft fractures, displaced fractures, conservative treatment

## Abstract

The treatment of displaced midshaft clavicle fractures (MCFs) is still controversial. The aims of our study were to evaluate clinical and radiological outcomes and complications of patients with displaced MCFs managed nonoperatively and to identify potential predictive factors of worse clinical outcomes. Seventy-five patients with displaced MCFs were enrolled and treated nonoperatively with a figure-of-eight bandage (F8-B). Initial shortening (IS) and displacement (ID) of fragments were radiographically evaluated at the time of diagnosis and immediately after F8-B application by residual shortening (RS) and displacement (RD). The clavicle shortening ratio was evaluated clinically at last follow-up. Functional outcomes were assessed using Constant (CS), q-DASH, DASH work and DASH sport scores. Cosmetic outcomes and rate of complications were evaluated. Good to very good mid-term clinical results were achieved by using the institutional treatment protocol. Multiple regression identified RS as an independent predictor of shoulder function, while RD affects fracture healing. These findings support the efficacy of our institutional protocol and thus could be useful for orthopedic surgeons during the decision-making process.

## 1. Introduction

Clavicle fractures represent 2.6–4% of all fractures on average [1], and up to 82% of them affect the clavicle midshaft [2]. A male predominance is reported, accounting for about 70% in young active male patients, while females are slightly more affected in elderly age [1,3,4]. These injuries commonly occur during athletic or recreational activities as the result of an axial force caused by a fall on the shoulder or on an outstretched hand, and traffic accidents or, less often, by a direct hit to the shoulder [5]. Midshaft clavicle fractures (MCFs) are among the most common upper extremity injuries managed by orthopedic trauma surgeons, and it is estimated that about half of all MCFs are displaced [6].

Clinical manifestations of clavicle fractures usually include pain and visible bone deformity as a consequence of the displacement of clavicle fragments [4].

Non-displaced MCFs are satisfactorily treated nonoperatively by sling immobilization, while the treatment of displaced fractures, which are the most frequent, is still under debate [7].

Acute displaced MCFs are traditionally managed successfully nonoperatively [8] showing good to very good results [3,9], while surgery becomes the treatment of choice in cases of failure of conservative treatment [10,11].

However, recent studies reporting higher nonunion rates after nonoperative treatment and an allegedly better clinical outcome after operative treatment, have led to a shift from nonoperative to operative treatment in the last 15–20 years [12,13,14]. Surgically treated patients, however, end up having to undergo second surgery for device removal procedures in more than 85% of cases [15].

To date, there are few absolute indications for early surgical fixation. Surgery is recommended in cases of open fractures, neurological deficiencies, compromised skin conditions, vascular injury, ipsilateral serial rib fractures, floating shoulder, widely displaced and comminuted fractures [8,16].

Nevertheless, current studies, including recent randomized controlled trials and meta-analyses, are still conflicting and fail to demonstrate the absolute superiority of surgical versus conservative management [6,14,17,18,19,20]. Several studies report better outcomes of surgery along with lower risk of nonunion compared to nonoperative management [18,21]. Conversely, other studies do not show differences in functional outcomes between conservative treatment and plate fixation of acute displaced MCFs, not only at one year of follow-up [10,13,14,22,23,24,25,26], but also after 24 weeks as well as after five years of follow-up [27]. Furthermore, surgical fixation is associated with complications in up to 29% of patients, such as wound infections, neurologic symptoms, frozen shoulder and implant-related problems [12].

Importantly, the identification of predictive factors of worse clinical outcome or non-union or symptomatic malunion is of great interest for the orthopedic surgeons as it would enable the identification of those patients at high risk of conservative treatment failure and help to avoid surgery overtreatment [28]. Jørgensen et al. published a systematic review reporting displacement as a likely risk factor of nonunion, while smoking, fracture comminution, age, gender and shortening were defined as doubtfully nonunion risk factors [28].

In a previous study published by our research group, predictive factors of delayed union and nonunion in adult patients with an MCF treated with a figure-of-eight bandage (F8-B) were investigated [29]. A residual displacement (RD) of 104%, assessed immediately after the application of the F8-B, was found to be a predictor that can help to differentiate patients who will heal, from patients who will develop delayed union and nonunion. Moreover, an RD of 140% was identified as an optimal cut-off point to distinguish between delayed union and nonunion [29]. Based on these findings, a treatment protocol for displaced MCFs was adopted in our clinic.

The objectives of this single-center study were (1) to evaluate clinical and radiological outcomes of patients with displaced MCFs managed nonoperatively following our institutional protocol and (2) to identify potential predictive factors of worse clinical outcomes.

## 2. Materials and Methods

### 2.1. Patient Selection

This study was designed as a single-center, retrospective, non-comparative case series, including patients affected by a displaced MCF between December 2015 and December 2018 and treated nonoperatively with an F8-B. All subjects participating in this experimental study received a thorough explanation of the risks and benefits of inclusion and gave their written informed consent to publish the data. This study was approved by the Institutional Ethics Committee (CESC code 189N/AO/21) and was performed in accordance with the ethical standards of the 1964 Declaration of Helsinki as revised in 2000 and those of Good Clinical Practice [30].

Inclusion criteria were: (1) patients with a traumatic, non-pathological, acute displaced MCF; (2) active patients between 18 and 65 years old; (3) at least 1-year clinical and radiographic follow-up; (4) RD ≤ 140% (see Section 2.3. Patient assessment) [29]. Exclusion criteria were: (1) ipsilateral neurological involvement; (2) patients receiving chemotherapy, radiotherapy and/or immunotherapy; (3) patients with a bilateral clavicle fracture; (4) patients with previous injury or surgery of the ipsilateral clavicle and/or shoulder; (5) patients who did not complete the entire follow-up program; (6) competitive athletes; (7) polytrauma patients; (8) RD > 140% (see Section 2.3. Patient assessment).

### 2.2. Treatment Protocol

**Figure 1:**
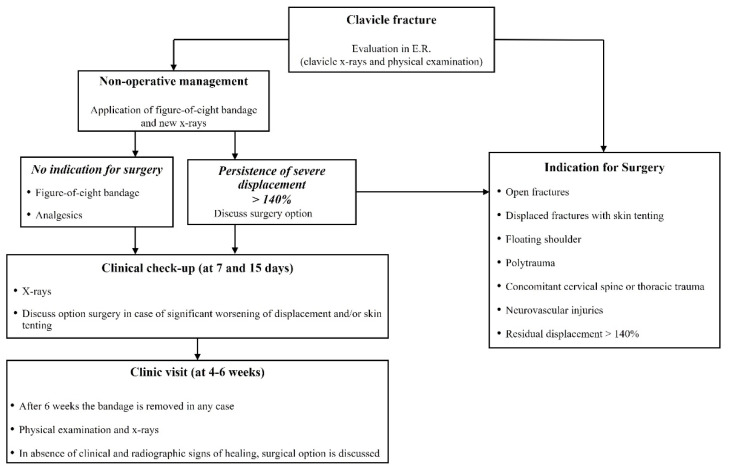
Institutional treatment protocol for displaced midshaft clavicle fracture of adult patients.

At our level-1 healthcare trauma center, a 1572-bed multi-disciplinary and multi-specialty regional university hospital, a highly standardized institutional treatment protocol, specific for patients with MCFs, was adopted (Figure 1).

**Figure 2:**
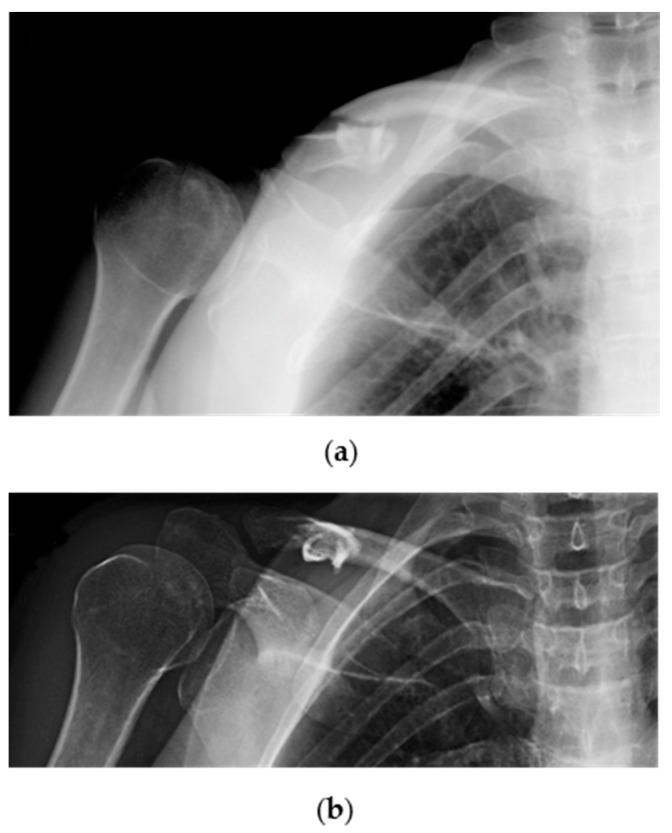
Radiographic images showing a traumatic displaced midshaft fracture of the right (dominant) clavicle in a 53-year-old female patient: (**a**) Cephalic tilt and; (**b**) anteroposterior X-ray views.

Patients were first evaluated by a trauma surgeon from our unit at the Emergency Room (ER) with plain X-rays (standard anteroposterior and 20° cephalic tilt views) (Figure 2: clinical case).

A careful physical examination was performed to evaluate functional impairment of the shoulder but also of the whole upper limb to exclude rare but possible associated injuries involving the brachial plexus or the subclavian vessels [4,16,31,32].

Other rare but potentially serious complications could involve the chest, such as the pneumothorax or hemothorax, which can be excluded with both thorough clinical and radiographic assessments [4,33,34,35,36,37,38].

Patients were referred to surgery in case of open fractures, displaced fractures with skin tenting, “floating shoulder”, polytrauma, concomitant cervical spine or thoracic trauma and neurovascular injuries. In all other cases, nonoperative management with an F8-B was suggested. Standard X-rays were repeated immediately after F8-B application in the ER to check the alignment of the fragments. Patients received thorough instructions on correct bandage use and positioning to avoid both decubitus ulcers in the axillary region and compression of the neurovascular bundle. All active movements of the shoulder were limited by the application of the F8-B; passive range of motion not above 90° forward flexion was permitted, while slight movements of the hand and the elbow (without load) were encouraged to prevent joint contractures and edema [3].

When severe RD persisted (>140%) after F8-B application, or when mechanical factors like soft tissue interposition, comminution or vertical fragments that impair reduction were suspected, surgical intervention was proposed [28].

Nonoperatively treated patients underwent both clinical and radiographic assessments at 7 days and 14 days after trauma to evaluate bandage tolerability and position and any possible worsening of fractures. When there was significant worsening of displacement and/or skin tenting, the option of surgery was discussed with the patient. The F8-B was maintained for 4–6 weeks, depending on fracture healing.

**Figure 3:**
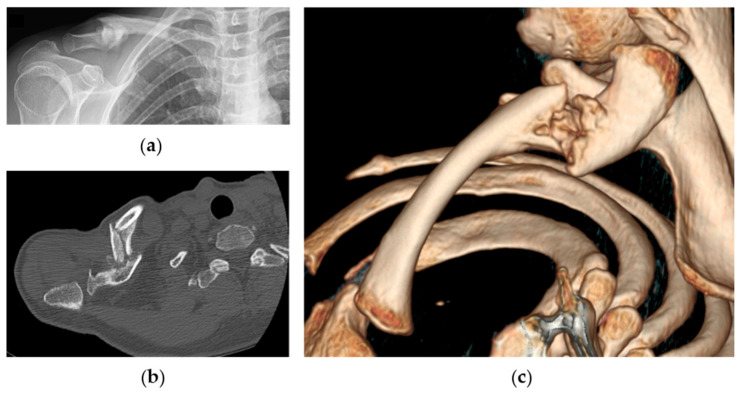
Radiographic images of the same patient showing right clavicle nonunion at 4-month follow-up: (**a**) anteroposterior X-ray view of the clavicle; (**b**) axial CT-scan evaluation of nonunion and; (**c**) 3D reconstruction.

In case of the absence of clinical and radiographic signs of healing after 6 weeks, including CT scan evaluation, the bandage was removed, and surgical reconstruction was discussed with the patient (Figure 3: clinical case).

### 2.3. Patient Assessment

Data collection was retrospectively performed by external and independent investigators, not involved in the patients’ treatment. Age, gender, body mass index (BMI), smoking status, presence of hypercholesterolemia and/or hypertension, mechanism of trauma, affected side and dominant limb involvement were recorded as baseline characteristics of the cohort.

Based on standard X-rays performed at patient admission at the ER of our hospital, radiographic fracture features were recorded as follows: fracture type (FT) according to the AO/OTA (Association for Osteosynthesis/Orthopedic Trauma Association) Classification [24]; initial shortening (IS) and residual shortening (RS), defined as the overlap of proximal and distal fragments and assessed as a percentage of the same clavicle length on a standard antero-posterior view, measured before and after the F8-B application; initial displacement (ID) and residual displacement (RD), measured as a percentage of the clavicle width at the fracture site on a 20° cephalic tilt view of the clavicle before and after the F8-B [29].

Clinical follow-up was performed on a weekly basis for two weeks after trauma and afterwards at 1-, 3-, 6-, and 12-months post-injury, while radiographic follow-up was prolonged until fracture union.

At the last follow-up visit, about one year after trauma, functional outcomes were measured by the Constant–Murley Score (CS) [25] and the Quick Disability of the Arm, Shoulder and Hand score (qDASH) [26]. CS consists of four items: pain; activities of daily living (ADL); range of motion (ROM); and strength. CS ranges from 0 to 100, indicating worst to optimum shoulder function. The qDASH score ranges from 0 to 100 with the latter representing the most disability and dysfunction; the optional qDASH work and sport modules were also used. 

In addition, time of return to work and return to sport or recreational activities were evaluated. A visual analogue scale (VAS) (range 0–10) was adopted to assess patient satisfaction of their functional status. The cosmetic outcome was assessed as a patient-reported outcome measure, asking patients if the treatment received had resulted in any negative effect on their quality of life.

The final clavicle shortening ratio compared with the contralateral clavicle was also assessed with a measuring tape at the last follow-up.

Finally, any complications were also recorded.

### 2.4. Nonoperative Rehabilitation Protocol

The F8-B was removed at 4–6 weeks. Then, the patients were trained to perform Codman exercises [39] and gradually, active shoulder movements as much as could be tolerated to achieve a full range of motion (ROM) over the next 3 or 4 weeks.

Lifting weight, heavy physical activity and contact sports were allowed only after complete clinical-radiological union of the fracture.

### 2.5. Statistical Analysis

Categorical variables were computed as percentages, while continuous parameters were expressed as means ± standard deviations. Normality of the data distribution was checked by means of a Shapiro–Wilk test. Univariate analyses were conducted using Student’s *t*-test and analysis of variance (ANOVA). Correlational analysis was conducted to shed light on the nature of the association between the various variables under study using the correlation coefficient assessment or its parametric version, depending on the normality of data distribution. Multivariate analysis of covariance (MANCOVA) was performed to identify the predictors of the outcome variables [40]. Partial eta squared was computed as effect size. MANCOVA assumptions (normal distribution of the dependent variables within groups; homogeneity of variance for each dependent variable and homogeneity of covariance for all the levels of the independent variable; linear relationship between the dependent variable and the covariates) were checked and met.

In case of statistical significance of a parameter, receiver operating characteristics (ROC) analysis was performed to quantitatively assess the effectiveness of a given classifier in terms of sensitivity and specificity. ROC analysis enables computing discrimination thresholds for variables of interest. We conducted ROC analysis by calculating the area under the curve (AUC) to obtain specific cut-off values. More specifically, the Youden J index was computed to identify the most acceptable trade-off in terms of sensitivity and specificity.

For all statistical analyses, a *p*-value less than 0.05 was considered statistically significant. All statistical analyses were carried out by means of the commercial software “Statistical Package for the Social Sciences” (SPSS, version 28.0 for Windows, IBM Corporation, Armonk, NY, USA) by an independent statistician.

## 3. Results

Seventy-five patients treated nonoperatively met the inclusion criteria and were enrolled in the study.

Demographic and clinical characteristics of the patients are reported in Table 1.

Mechanisms of trauma were a bike fall for twenty-nine cases (38.7%), a motorcycle trauma for twenty-one cases (28.0%), low energy traumas such as a sports injury for sixteen patients (21.3%) and a simple fall in nine patients (12.0%).

The radiographic parameters of enrolled patients are reported in Table 2.

The mean IS and RS were 5.4 ± 4.6% and 3.4 ± 3.6%, while mean ID and RD were 113 ± 43.4% and 91.8 ± 30.8 %, respectively. Mean follow-up time was 27.5 ± 7.5 months.

A mean total CS of 96.8 ± 5.6 and total qDASH of 4.2 ± 6.3 were recorded (Table 3).

Mean time of return to work was 2.5 ± 1.1 months, while mean time of return to sports or recreational activities was 4.1 ± 1.8 months. Mean patient satisfaction was 7.6 ± 1.0. Thirty patients (40%) had cosmetic problems. The mean shortening ratio at last follow-up was 3.5 ± 3.5% (Table 3).

Regarding complications, refractures and delayed healing were reported in five and eleven patients, respectively.

All cases of refracture occurred in clinically and radiographically healed patients, after a forceful trauma, and at least four months after the first trauma. All cases were subsequently treated surgically. Conversely, delayed healing is referred to fractures not healed clinically and radiographically within three months from the trauma, but healed later within five months. The mean RD of delayed healed patients was 120.4% ± 15.8.

None of the patients suffered nonunion.

Correlations between clinical outcomes and radiological features were evaluated and reported in Table S1. Total CS and its subscales showed an inverse correlation with IS, RS and shortening ratio with higher values corresponding to lower total CS and subscale values. qDASH score, qDASH Work and qDASH sport correlated with IS, RS and shortening ratio, with higher values corresponding to greater qDASH values.

Return to work correlated in terms of ID, IS and shortening ratio with higher values corresponding to higher values of return to work.

No correlations were found between radiological features and return to sport.

### Potential Predictive Factors

Multivariate analysis of covariance (MANCOVA) was performed to identify predictors of the outcome variables total CS and the qDASH score and their subscales. With the MANCOVA, RS resulted a statistically significant predictor (Table 4). All data of MANCOVA analysis are described in Table S2.

Age, BMI and smoking were not statistically significant predictors (data not shown).

An ROC curve analysis was performed to identify cut-off points for radiological features and functional outcomes.

**Figure 4:**
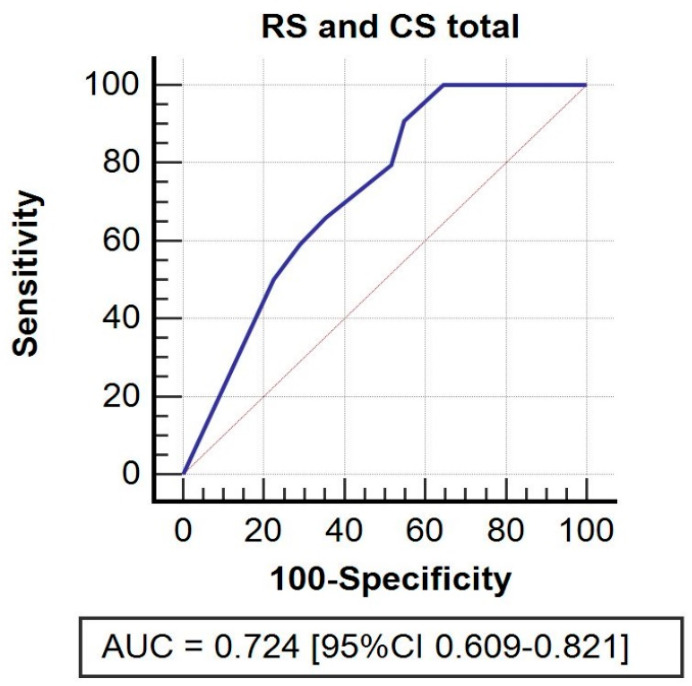
ROC curve. Receiver operating characteristic (ROC) curve for residual shortening and its impact on total CS.

RS (B coefficient = −1.55, *p* < 0.001; cut-off = 5, sensitivity 90.91%, specificity 45.16%) impacted total CS with lower RS values corresponding to higher total CS (Figure 4). 

RS impacted pain (B coefficient = −0.21, *p* = 0.030; cut-off = 5, sensitivity 87.10%, specificity 76.92%), with lower values of RS correlating with greater pain, as well as ROM (B coefficient = −0.27, *p* = 0.017; cut-off = 5, sensitivity 85.00%, specificity 60.00%) with a similar relationship, and ADL (B coefficient t= −0.19, *p* = 0.045; cut-off = 6, sensitivity 93.94%, specificity 77.78%), and in the latter case together with shortening ratio (B = −0.03, *p* = 0.021; cut-off = 6.7, sensitivity 95.45%, specificity 88.89%), with lower values of RS and shortening ratio corresponding to higher values of ADL.

RS (B coefficient = −0.80, *p* < 0.001; cut-off = 6, sensitivity 94.34%, specificity 36.36%) impacted strength. Lower RS values corresponded to greater strength values (Figure S1).

There was no significant determinant for qDASH, qDASH work and qDASH sport. 

RS (B coefficient = 0.22, *p* = 0.012; cut-off = 2, sensitivity 59.32%, specificity 68.75%) impacted return to work, with higher RS values corresponding to later return to work.

## 4. Discussion

The objective of the present study was first to evaluate clinical and radiological outcomes of patients with displaced MCFs managed nonoperatively with an F8-B according to the protocol developed by our institution, which aimed to decrease nonunion rates, and secondly, to identify predictive factors of worse clinical outcomes. In our cohort, displaced MCFs mostly affected young male patients. Most of the patients were not overweight, had normal blood pressure and levels of LDL, all considered risk factors of developing nonunion [18,41,42,43]. Although almost 50% of patients were active smokers, smoking did not affect the functional outcomes evaluated in this study.

Most of the patients showed good clinical outcomes at their last follow-up appointment, both in terms of total CS and its subscales, and qDASH, qDASH work and qDASH sport.

In our study, according to Ziegler et al. [44] and Subramanyam et al. [18], who divided CS into four categories (very good 86–100, good 71–85, fair 56–70, poor <56), CS was very good in 93.3% of the patients and good in 6.7% of the patients, thus confirming the effective results of nonoperative management of displaced MCFs at medium-term follow-up [14,18,19]. 

These data were also supported by the good results of total qDASH (4.2 ± 6.3 points), DASH work (3.5 ± 9.1 points) and DASH sport (5.2 ± 11.8 points), highlighting the presence of low residual disability at follow-up as reported by Woltz et al., 2017 and Amer et al., 2020 [14,19].

Correlations between radiological features evaluated before and after the F8-B and clinical outcomes were also evaluated. More specifically, an inverse correlation was found between IS, RS and the shortening ratio and total CS and its subscales, confirming that lower values of shortening lead to higher values of CS, and therefore better functional outcomes.

These data were also confirmed with total qDASH, qDASH work and qDASH sport that directly correlate with IS, RS and shortening ratio with higher values of qDASH (higher upper limb disability) corresponding to higher values of shortening. Return to work was directly correlated with IS and RS, and the data obtained in our study are in line with what is reported in the recent literature [45].

VAS satisfaction showed good results with most of the patients satisfied. These data are similar to those of Woltz et al. (2018), even if some authors reported higher patient satisfaction in cases of early surgical management [46,47]. One of the most frequent reasons for patient dissatisfaction with the result of conservative treatment is poor cosmetic appearance, which emerged particularly among women [48]. Hence, cosmetic dissatisfaction was reported by 40% of our patients, a percentage lower than that reported in the literature [13].

Regarding complications, all the patients recovered with the conservative treatment and no nonunion was found, thus confirming the efficacy of the F8-B applied according to our institutional protocol, developed based on the findings of our previous analysis (including patients with RD ≤ 140%) [29]. This confirms the importance of fracture morphology with regard to healing.

Therefore, the absence of nonunion is an important result considering that the nonunion rate reported in the literature ranged between 5% and 20% after nonoperative treatment [19,20,42,49,50].

The 11 patients with delayed healing displayed an RD > 104% but less than 140%, in agreement with our previous study [29]. This result points out the importance not only of treatment selection but also of an appropriate follow-up of these patients that are at higher risk of having delayed healing with conservative treatment. In this context, our institutional protocol could be considered a useful guideline to identify how much displacement can be considered acceptable for nonoperative management (RD ≤ 140%), and when displaced fractures should be treated surgically.

Multivariate analysis was performed to identify predictive factors of worse clinical outcomes. RS results showed it to be a radiological predictor of worse shoulder function, as expressed by total CS with a cut-off value of five and its subscales: pain; ADL; ROM; and strength. Shortening ratio is also a predictive factor of worse ADL, with lower values of shortening ratio corresponding to higher values of ADL. To the best of our knowledge, no studies evaluated the RD and RS after F8-B application, hindering the comparison with other studies. The current literature focuses only on ID and IS [48,51,52,53]. Jones et al. reported that ID is better than IS as a predictor of worse outcomes [54]. Several studies found no association between IS and functional outcomes in agreement with our data outcomes [51,52,55]. Conversely, other studies reported an association between IS and worse outcomes [12,18,39,53,56]. Importantly, two systematic reviews analyzed in detail the impact of IS on the clinical outcomes, concluding that, actually, the published studies do not support an association between the two [49,57]. It should also be pointed out that different methods of measuring clavicle shortening are applied, different immobilization methods are used, as well as different follow-up times, making a comparison between studies difficult.

The present study has several points of weakness and strength. Its main limitations are the retrospective design and the relatively small sample size of a single center. This limitation was due to the choice to enroll only patients who had completed the entire clinical and radiological follow-up and to the strict inclusion criteria. Another limit is the lack of a control group (for example one treated by a simple arm sling), since the study was designed as non-comparative. Furthermore, it could be argued that shoulder position during radiography also has a substantial effect on the length of the fractured clavicle, altering its measurements. However, in our study, the patient was facing toward the radiography film in a standardized way before and after F8-B application to improve the fracture position and to reduce the risks of radiographic bias.

Regarding strengths, our study cohort was a consecutive series of patients treated in our center for an MCF according to our specific, standardized, institutional protocol and followed until fracture healing. Furthermore, contrary to most publications on this subject, displacement and shortening of the fracture were expressed in percentage with respect to the length of the ipsilateral clavicle, after using standardized radiological method to evaluate both displacement and shortening as recommended. This relative measure can be applied to each subject regardless of his or her body characteristics, unlike the expression in length.

To the best of our knowledge, this is the first study that assesses functional outcomes and standardized radiographic aspects (before and after the application of the F8-B) at the time of evaluation in the emergency room, and when evaluating the shortening ratio at last follow-up as well.

Further large-scale, prospective, randomized controlled trials are necessary to better identify those patients in stratified high-risk groups who would be more likely to suffer nonunion and would benefit from early surgery.

## 5. Conclusions

In conclusion, good to very good mid-term clinical results were obtained managing displaced MCFs of adult patients with conservative treatment according to our institutional protocol. Only a few cases of suboptimal functional scores were recorded, which were attributable to residual clavicular shortening, when severe. In addition, while residual displacement was found to have an impact on fracture healing, residual shortening was a predictor of functional clinical outcomes.

We believe that the findings of this study could be useful for orthopedic surgeons and the treatment should be accurately discussed with patients, explaining the risks and benefits of each therapeutic approach. Finally, the treatment option should be carefully personalized, considering each patient’s psycho-physical features, activity level and expectations.

## Figures and Tables

**Table 1:** Demographic and clinical characteristics of overall patients.

Variable	Patients Enrolled *n* = 75
Age, mean (SD)	42.8 (13.7)
Gender, number (%)	
male	62 (82.7)
female	13 (17.3)
BMI, mean (SD)	24.1 (2.3)
Smoking status, number (%)	
active	33 (44.0)
inactive	42 (56.0)
Hypercholesterolemia, number (%)	
LDL ≥ 240 mg/dL	7 (9.3)
LDL < 240 md/dL	68 (90.7)
Hypertension, number (%)	
Systolic ≥ 130, Diastolic ≥ 80	17 (22.7)
Systolic < 130, Diastolic < 80	58 (77.3)
Type of trauma, number (%)	
Bike Fall	29 (38.7)
Motorcycle trauma	21 (28.0)
Sport injury	16 (21.3)
Simple fall	9 (12.0)
Dominant side involved,	
number (%)	31 (41.3)

SD = Standard Deviation; BMI = Body Mass Index; LDL = Low Density Lipoproteins. (Hypercholesterolemia: LDL ≥ 240 mg/dL).

**Table 2:** Radiological characteristics of patients.

Variable	Patients Enrolled *n* = 75
Type of fracture, number (%)	
A1	5 (6.7)
A2	20 (26.7)
A3	6 (8.0)
B1	3 (4.0)
B2	13 (17.3)
B3	28 (37.3)
Initial shortening (%), mean (SD)	5.4 (4.6)
Residual shortening (%), mean (SD)	3.4 (3.6)
Initial displacement (%), mean (SD)	113 (43.4)
Residual displacement (%), mean (SD)	91.8 (30.8)

SD = Standard Deviation.

**Table 3:** Clinical outcomes of patients at follow-up (Mean follow-up time was 27.5 ± 7.5 months).

Outcomes	Patients Enrolled *n* = 75
Constant score, mean (SD)	
Total	96.8 (5.6)
Pain subscale	14.6 (1.2)
Activity Daily Living subscale	19.6 (1.2)
Range of movement subscale	39.3 (1.5)
Strength subscale	23.3 (3.1)
qDASH score, mean (SD)	
Total	4.2 (6.3)
Work	3.5 (9.1)
Sport	5.2 (11.8)
Return to work (months), mean (SD)	2.5 (1.1)
Return to sport (months), mean (SD)	4.1 (1.8)
VAS satisfaction, mean (SD)	7.6 (1.0)
Cosmetic problem, number (%)	30 (40)
Shortening ratio (%), mean (SD)	3.5 (3.5)

SD = Standard Deviation; qDASH = Quick Disabilities of the Arm, Shoulder and Hand; VAS = Visual Analogic Scale.

**Table 4:** Radiological predictors of shoulder function (MANCOVA).

Dependent Variable	Parameter	B	Std. Error	t	*p*-Value	95% Confidence Interval				
						Lower Bound	Upper Bound	Partial Eta Squared	Noncent. Parameter	Observed Power
CS total	Intercept	99.129	7.412	13.374	<.001	84.241	114.017	0.782	13.374	1.000
	ID	−0.020	0.017	−1.180	0.244	−0.054	0.014	0.027	1.180	0.212
	RD	0.016	0.025	0.637	0.527	−0.034	0.066	0.008	0.637	0.096
	IS	0.510	0.265	1.922	0.060	−0.023	1.042	0.069	1.922	0.470
	RS	−1.554	0.335	−4.637	**<0.001**	−2.228	−0.881	0.301	4.637	0.995
	Shortening ratio	−0.069	0.047	−1.461	0.150	−0.164	0.026	0.041	1.461	0.300
Pain	Intercept	10.282	2.068	4.972	<0.001	6.128	14.435	0.331	4.972	0.998
	ID	0.001	0.005	0.276	0.784	−0.008	0.011	0.002	0.276	0.058
	RD	0.008	0.007	1.079	0.286	−0.006	0.022	0.023	1.079	0.185
	IS	0.024	0.074	0.318	0.751	−0.125	0.172	0.002	0.318	0.061
	RS	−0.209	0.094	−2.235	**0.030**	−0.397	−0.021	0.091	2.235	0.592
	Shortening ratio	−0.004	0.013	−0.328	0.745	−0.031	0.022	0.002	0.328	0.062
ADL	Intercept	17.816	2.081	8.559	<0.001	13.635	21.997	0.594	8.559	1.000
	ID	−0.001	0.005	−0.315	0.754	−0.011	0.008	0.002	0.315	0.061
	RD	0.005	0.007	0.665	0.509	−0.009	0.019	0.009	0.665	0.100
	IS	0.048	0.074	0.647	0.521	−0.101	0.198	0.008	0.647	0.097
	RS	−0.193	0.094	−2.054	**0.045**	−0.382	−0.004	0.078	2.054	0.522
	Shortening ratio	−.032	0.013	−2.375	**0.021**	−0.058	−0.005	0.101	2.375	0.644
ROM	Intercept	45.954	2.442	18.816	<0.001	41.049	50.860	0.876	18.816	1.000
	ID	−0.006	0.006	−1.079	0.286	−0.017	0.005	0.023	1.079	0.185
	RD	−0.001	0.008	−.146	0.885	−0.018	0.015	0.000	0.146	0.052
	IS	0.095	0.087	1.090	0.281	−0.080	0.271	0.023	1.090	0.188
	RS	−0.272	0.110	−2.466	**0.017**	−0.494	−0.051	0.108	2.466	0.677
	Shortening ratio	−0.030	0.016	−1.905	0.063	−0.061	0.002	0.068	1.905	0.464
Strength	Intercept	25.679	4.299	5.974	<0.001	17.045	34.313	0.416	5.974	1.000
	ID	−0.015	0.010	−1.551	0.127	−0.035	0.004	0.046	1.551	0.331
	RD	0.006	0.015	0.397	0.693	−0.023	0.035	0.003	0.397	0.068
	IS	0.340	0.154	2.209	**0.032**	0.031	0.648	0.089	2.209	0.582
	RS	−0.799	0.194	−4.110	**<0.001**	−1.190	−0.409	0.253	4.110	0.981
	Shortening ratio	−.030	0.027	−1.082	0.284	−0.085	0.025	0.023	1.082	0.186

CS = Constant Score; ID = Initial Displacement; RD = Residual Displacement; IS = Initial Shortening; RS = Residual Shortening. Statistically significant *p*-value are bolded.

## Data Availability

Data are available contacting the corresponding authors on reasonable request.

## Supplementary Materials

The following are available online at https://www.mdpi.com/article/10.3390/jpm12050759/s1, Table S1: Correlations between clinical outcomes and radiological features; Table S2: Radiological predictors of clinical outcomes (MANCOVA); Figure S1: ROC curve for residual displacement and its impact on CS subscales: pain, ADL, ROM and strength.
