# Supplementary material for: Management of Displaced Midshaft Clavicle Fractures with Figure-of-Eight Bandage: The Impact of Residual Shortening on Shoulder Function
Source: J Pers Med. 2022 May 7;12(5):759. doi: 10.3390/jpm12050759 (PMC9145303; doi:10.3390/jpm12050759)
Supplement: Supplementary file 1 [file jpm-12-00759-s001.zip › jpm-1660411-supplementary.pdf]

Table S1: Correlations between clinical outcomes and radiological features.

Variable	Initial displacement	Residual displacement	Initial shortening	Residual shortening	Shortening ratio
Constant score, Total	ns	ns	- 0.432, <0.001	- 0.500, <0.001	- 0.663, <0.001
Pain	ns	ns	- 0.437, <0.001	- 0.475, <0.001	- 0.482, <0.001
ADL	ns	ns	- 0.373, 0.001	- 0.398, <0.001	- 0.454, <0.001
ROM	ns	ns	- 0.401, <0.001	- 0.460, <0.001	- 0.500, <0.001
Strength	ns	ns	- 0.294, 0.010	- 0.363, 0.001	- 0.582, <0.001
qDASH score, Total	ns	ns	0.348, 0.002	0.411, < 0.001	0.581, < 0.001
qDASH Work	ns	ns	0.363, 0.001	0.440, < 0.001	0.482, < 0.001
qDASH Sport	ns	ns	0.322, 0.005	0.347, 0.001	0.364, 0.001
Return to work (months)	0.271, 0.019	ns	0.323, 0.005	ns	0.333, 0.004
Return to sport (months)	ns	ns	ns	ns	ns
VAS satisfaction	- 0.247, 0.033	ns	ns	ns	- 0.292, 0.014

qDASH = Quick Disabilities of the Arm, Shoulder, and Hand; VAS = Visual Analogic Scale.
All data are expressed as R, p-value. ns = not significant

Table S2: Radiological predictors of clinical outcomes (MANCOVA).

Dependent Variable	Parameter	B	Std. Error	t	Sig.	95% Confidence Interval Lower Bound	95% Confidence Interval Upper Bound	Partial Eta Squared	Noncent. Parameter	Observed Power
CS total	Intercept	99.129	7.412	13.374	<0.001	84.241	114.017	0.782	13.374	1.000
	ID	-0.020	0.017	-1.180	0.244	-0.054	0.014	0.027	1.180	0.212
	RD	0.016	0.025	0.637	0.527	-0.034	0.066	0.008	0.637	0.096
	IS	0.510	0.265	1.922	0.060	-0.023	1.042	0.069	1.922	0.470
	RS	-1.554	0.335	-4.637	<0.001	-2.228	-0.881	0.301	4.637	0.995
	Shortening ratio	-0.069	0.047	-1.461	0.150	-0.164	0.026	0.041	1.461	0.300
Pain	Intercept	10.282	2.068	4.972	<0.001	6.128	14.435	0.331	4.972	0.998
	ID	0.001	0.005	0.276	0.784	-0.008	0.011	0.002	0.276	0.058
	RD	0.008	0.007	1.079	0.286	-0.006	0.022	0.023	1.079	0.185
	IS	0.024	0.074	0.318	0.751	-0.125	0.172	0.002	0.318	0.061
	RS	-0.209	0.094	-2.235	0.030	-0.397	-0.021	0.091	2.235	0.592
	Shortening ratio	-0.004	0.013	-0.328	0.745	-0.031	0.022	0.002	0.328	0.062
ADL	Intercept	17.816	2.081	8.559	<0.001	13.635	21.997	0.594	8.559	1.000
	ID	-0.001	0.005	-0.315	0.754	-0.011	0.008	0.002	0.315	0.061
	RD	0.005	0.007	0.665	0.509	-0.009	0.019	0.009	0.665	0.100
	IS	0.048	0.074	0.647	0.521	-0.101	0.198	0.008	0.647	0.097
	RS	-0.193	0.094	-2.054	0.045	-0.382	-0.004	0.078	2.054	0.522
	Shortening ratio	-0.032	0.013	-2.375	0.021	-0.058	-0.005	0.101	2.375	0.644
ROM	Intercept	45.954	2.442	18.816	<0.001	41.049	50.860	0.876	18.816	1.000
	ID	-0.006	0.006	-1.079	0.286	-0.017	0.005	0.023	1.079	0.185
	RD	-0.001	0.008	-0.146	0.885	-0.018	0.015	0.000	0.146	0.052
	IS	0.095	0.087	1.090	0.281	-0.080	0.271	0.023	1.090	0.188
	RS	-0.272	0.110	-2.466	0.017	-0.494	-0.051	0.108	2.466	0.677
	Shortening ratio	-0.030	0.016	-1.905	0.063	-0.061	0.002	0.068	1.905	0.464
Strenght	Intercept	25.679	4.299	5.974	<0.001	17.045	34.313	0.416	5.974	1.000
	ID	-0.015	0.010	-1.551	0.127	-0.035	0.004	0.046	1.551	0.331
	RD	0.006	0.015	0.397	0.693	-0.023	0.035	0.003	0.397	0.068
	IS	0.340	0.154	2.209	0.032	0.031	0.648	0.089	2.209	0.582
	RS	-0.799	0.194	-4.110	<0.001	-1.190	-0.409	0.253	4.110	0.981
	Shortening ratio	-0.030	0.027	-1.082	0.284	-0.085	0.025	0.023	1.082	0.186
qDASH	Intercept	279.396	579.908	0.482	0.632	-885.383	1444.175	0.005	0.482	0.076
	ID	-1.143	1.322	-0.864	0.392	-3.799	1.513	0.015	0.864	0.135
	RD	-0.031	1.957	-0.016	0.987	-3.962	3.900	0.000	0.016	0.050
	IS	4.796	20.742	0.231	0.818	-36.864	46.457	0.001	0.231	0.056
	RS	22.984	26.228	0.876	0.385	-29.697	75.666	0.015	0.876	0.138
	Shortening ratio	-2.211	3.696	-0.598	0.552	-9.634	5.212	0.007	0.598	0.090

qDASH_Work	Intercept	-794.927	1353.664	-0.587	0.560	-3513.841	1923.987	0.007	0.587	0.089
	ID	-0.592	3.087	-0.192	0.849	-6.792	5.608	0.001	0.192	0.054
	RD	1.207	4.569	0.264	0.793	-7.970	10.383	0.001	0.264	0.058
	IS	-3.290	48.417	-0.068	0.946	-100.537	93.958	0.000	0.068	0.051
	RS	38.913	61.224	0.636	0.528	-84.060	161.886	0.008	0.636	0.096
	Shortening ratio	-3.790	8.627	-0.439	0.662	-21.118	13.538	0.004	0.439	0.072
qDASH_Sport	Intercept	2138.958	2003.231	1.068	0.291	-1884.650	6162.566	0.022	1.068	0.182
	ID	-4.230	4.568	-0.926	0.359	-13.405	4.946	0.017	0.926	0.149
	RD	-1.437	6.761	-0.213	0.833	-15.017	12.143	0.001	0.213	0.055
	IS	-8.645	71.650	-0.121	0.904	-152.558	135.267	0.000	0.121	0.052
	RS	120.019	90.604	1.325	0.191	-61.964	302.001	0.034	1.325	0.255
	Shortening ratio	-7.170	12.767	-0.562	0.577	-32.813	18.473	0.006	0.562	0.085
Work_months	Intercept	1.260	1.849	0.682	0.499	-2.453	4.974	0.009	0.682	0.103
	ID	-0.003	0.004	-0.610	0.545	-0.011	0.006	0.007	0.610	0.092
	RD	0.005	0.006	0.761	0.450	-0.008	0.017	0.011	0.761	0.116
	IS	-0.096	0.066	-1.453	0.152	-0.229	0.037	0.041	1.453	0.297
	RS	0.217	0.084	2.600	0.012	0.049	0.385	0.119	2.600	0.722
	Shortening ratio	0.010	0.012	0.853	0.398	-0.014	0.034	0.014	0.853	0.133
Sport_months	Intercept	3.044	3.364	0.905	0.370	-3.714	9.801	0.016	0.905	0.144
	ID	-0.010	0.008	-1.285	0.205	-0.025	0.006	0.032	1.285	0.243
	RD	0.004	0.011	0.391	0.697	-0.018	0.027	0.003	0.391	0.067
	IS	-0.032	0.120	-0.262	0.794	-0.273	0.210	0.001	0.262	0.058
	RS	0.144	0.152	0.947	0.348	-0.161	0.450	0.018	0.947	0.153
	Shortening ratio	0.004	0.021	0.209	0.836	-0.039	0.048	0.001	0.209	0.055

CS=Constant Score; ID=Initial Displacement; RD=Residual Displacement; IS=Initial Shortening; RS=Residual Shortening; qDASH = Quick Disabilities of the Arm, Shoulder, and Hand.

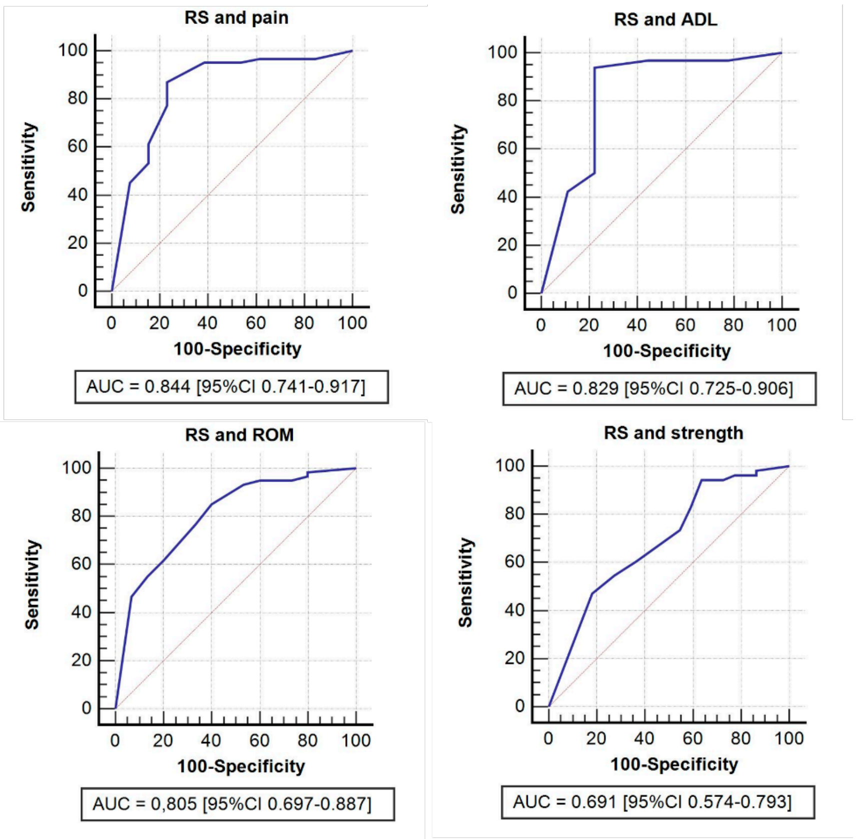

Figure S1. ROC curves for residual shortening and its impact CS subscales: pain, ADL, ROM, and strength.
